# Outcomes of Acute Pancreatitis in Patients With and Without Liver Cirrhosis: A Retrospective Analysis

**DOI:** 10.7759/cureus.78933

**Published:** 2025-02-13

**Authors:** Ahmed Ali Aziz, Muhammad Ali Aziz, Muhammad Amir, Rehan Shah, Ijlal Akbar Ali

**Affiliations:** 1 Internal Medicine, INTEGRIS Health Baptist Medical Center, Oklahoma City, USA; 2 Internal Medicine, University of Kentucky College of Medicine, Lexington, USA; 3 Transplant Hepatology, INTEGRIS Health Baptist Medical Center, Oklahoma City, USA; 4 Internal Medicine, Bayonne Medical Center, Bayonne, USA; 5 Digestive Diseases and Nutrition, University of Oklahoma Health Sciences Center, Oklahoma City, USA

**Keywords:** acute pancreatitis, chronic liver failure, cirrhosis of the liver, morbidity and mortality, national inpatient sample database

## Abstract

Background

Acute pancreatitis (AP) is a common cause of hospitalization in the United States. Our study aimed to investigate the impact of liver cirrhosis (LC) on the outcomes of AP in adult patients hospitalized with AP.

Methods

We performed a retrospective study of adult patients with AP utilizing the Nationwide Inpatient Sample (NIS) database from 2020 to 2022. We compared AP outcomes in patients with and without LC. The primary outcome was all-cause inpatient mortality. Secondary outcomes were the length of stay (LOS), healthcare cost utilization adjusted to 2022, the incidence of acute renal failure (ARF), sepsis, shock, venous thromboembolism (VTE), and the need for intensive care unit (ICU) admission. Statistical analyses were performed using STATA, version 16.1 (StataCorp., College Station, Texas, USA). A multivariate logistic regression analysis was conducted to assess if gender was an independent predictor for these outcomes and to adjust for any confounders.

Results

From 2020 to 2022, 738,139 adult patients underwent AP admissions. Among the patients, 723,959 had AP without LC, and 14,180 had AP with LC. The mean age for patients with and without LC was the same, 50.9 years. Patients without LC had a higher prevalence of cerebrovascular accident (CVA) and obesity. Patients with LC had a higher prevalence of congestive heart failure (CHF), diabetes mellitus type 2 (DM2), diabetes mellitus type 1 (DM1), chronic kidney disease (CKD), and smoking/tobacco use. We found that AP patients with LC had a significantly higher likelihood of in-hospital mortality (aOR: 1.74, 95% CI: 1.22-2.42, P < 0.01), longer LOS (+ 0.46 days, 95% CI: 0.26-0.67, P < 0.01), higher healthcare utilization cost (+ $4163, 95% CI: $1530.9-$6796.0, P < 0.01), shock (aOR: 1.87, 95% CI: 1.42-2.46, P < 0.01), ARF (aOR: 1.44, 95% CI: 1.30-1.59, P < 0.01), ICU admission (aOR: 1.67, 95% CI: 1.38-1.91, P < 0.01), and acute VTE (aOR: 1.65, 95% CI: 1.30-2.11, P < 0.01).

Conclusions

We found that patients with AP who concomitantly had LC had significantly poor clinical outcomes, including higher mortality, ARF, shock, ICU admission, VTE, LOS, and total hospitalization charges as compared to AP patients who did not have LC. Our study highlights that cirrhotic patients with AP have poor inpatient hospital outcomes and need aggressive treatment to prevent morbidity and mortality.

## Introduction

Acute pancreatitis (AP) is one of the most common gastrointestinal (GI) diseases and is the third most common cause of gastroenterology-related hospitalization in the United States [1]. Literature shows that the incidence of AP is increasing in the USA [2,3]. Liver cirrhosis (LC) is also a very prevalent GI-related disease. Estimates show that around 800 million people suffer from cirrhosis annually, with two million deaths per year [4]. Both AP and LC have a significant cost-related burden on the U.S. healthcare system due to their increased incidence and prevalence, respectively [5]. The rising prevalence of obesity and alcohol consumption in the general population has also increased the incidence of AP and the prevalence of liver cirrhosis [6]. One can notice a significant overlap between risk factors and causes of AP and LC. Treatment is often complex as AP treatment involves fluid resuscitation while patients with LC are usually fluid overloaded. This poses a significant dilemma for the treatment of AP in LC [7-9]. Only limited studies report the epidemiologic relationship between AP and LC and the outcomes of AP in patients with LC. We conducted a study to investigate the outcomes of AP in patients with and without LC and to study the epidemiologic relationship between AP and LC.

## Materials and methods

Data source

The study was conducted at INTEGRIS Health Baptist Medical Center, Oklahoma City. We performed a retrospective analysis of the National Inpatient Sample (NIS) database from 2020 to 2022. NIS is the largest public database maintained by the Healthcare Cost and Utilization Project (HCUP). It contains data from 48 states and more than 98% of the U.S. population. It is sampled from the State Inpatient Databases and contains data on roughly seven million hospitalizations each year. The database is a 20% stratified sample of all hospital admissions in the United States and provides reliable inpatient estimates of comorbidity and disease prevalence. Each discharge from the resultant data is then weighted (where the weight equals the total number of discharges from all acute care hospitals in the United States divided by the number of discharges included in the 20% sample) to make the NIS nationally representative. When weighted, the NIS data estimates around 35 million hospitalizations across the US per year [10]. NIS contains information about patient demographics, admission profile, discharge diagnosis, length of stay (LOS), and hospital charges. We used the International Classification of Diseases (ICD-10), World Health Organization (WHO) (https://www.cdc.gov/nchs/icd/icd-10-cm/index.html) codes to conduct this study [11].

Study population

We used the ICD-10 code (K85) to identify all patients with a primary discharge diagnosis of AP. We then used the ICD-10 code (K74) to identify all patients with a secondary diagnosis of LC. We then divided the patients with the primary diagnosis of AP into patients who had liver cirrhosis as a secondary diagnosis and called this group “AP with LC” and into patients who did not have liver cirrhosis as a secondary diagnosis and called this group “AP without LC.”. Patients were excluded if they were <18 years old, had an elective admission, were transferred from other facilities, or died on the day of admission. Our study was exempt from the Institutional Review Board approval as NIS is a publicly available administrative database and contains de-identified data.

Study variables

We extracted age, biological sex, income quartile, primary payer (Medicare, Medicaid, private, self-pay), hospital location, hospital region, hospital bed size, hospital teaching status, and race (White, Black, Hispanic, Asian/Pacific Islander, Native American, Other). NIS classifies any race that is not White, Black, Hispanic, Asian/Pacific Islander, or Native American as Other. The burden of comorbidities was assessed using the Charlson comorbidity index (CCI).

Study outcomes

The primary outcome was in-hospital all-cause mortality. Secondary outcomes included mean LOS, mean total hospitalization charges adjusted to the year 2022, the incidence of acute renal failure (ARF), sepsis, shock, acute venous thromboembolism (VTE) and need for intensive care unit (ICU) admission.

Statistical analysis

We used STATA statistical software, version 16.1 (StataCorp., College Station, Texas, USA), to perform statistical analysis. The software allows using NIS to produce nationally representative results. Proportions were compared using the chi-square test. Continuous variables were compared using the student t-test. Univariate analysis was used to assess the association between each variable and the outcomes. Variables with a p-value of less than 0.2 were included in multivariate regression analysis. Multivariate regression analysis was then used to calculate outcomes and to remove the effect of possible confounders. We adjusted for possible confounders including age, race, Charlson disease severity index, hospital bed size, hospital location, hospital teaching status, and patient’s insurance status. Logistic regression was used for binary outcomes, and linear regression was used for continuous outcomes. A P-value < 0.05 was determined to be considered statistically significant for all outcomes.

## Results

Patient and hospital characteristics

The NIS database for 2020-2022 contained over 98 million weighted hospital discharges, of which 738,139 had a principal discharge diagnosis of acute pancreatitis. Of the patients, 723,959 had AP without LC, and 14,180 had AP with LC. The mean age of AP patients with and without LC was the same, 50.9 years. Patient and hospital characteristics of AP patients with and without LC are presented in Table 1. The percentage subgroup etiology of AP was nearly the same in both groups. Medicare was the primary payer for AP patients with LC (39.57%), while private insurance was the primary payer for most AP patients without LC (33.96%). Patients with LC had a higher overall CCI. For both groups, most patients had a median income of $1-$51,999 (34.90% with LC and 31.60% without LC) and were admitted to urban teaching hospitals (73.27% with LC and 68.93% without LC). Caucasian individuals were the most prevalent race.

**Table 1:** Patient and hospital characteristics of acute pancreatitis (AP) patients with and without liver cirrhosis (LC) AP: acute pancreatitis; CVA: cerebrovascular accident

Variable	AP without liver cirrhosis	AP with liver cirrhosis	P-value
Proportion	723,959 (98%)	14,180 (2%)	
Gender			< 0.01
Female	329,184 (45.47%)	6,061 (42.74%)	
Male	394,775 (54.53%)	8,119 (57.26%)	
Mean age (years)	50.9	50.9	< 0.01
Etiology of AP			< 0.01
Idiopathic	286,69 (3.96%)	386 (2.72 %)	
Biliary	123,145 (17.01%)	2,360 (16.64 %)	
Alcoholic	224,717 (31.04%)	3,674 (25.91%)	
Drug-induced	10,715 (1.48%)	180 (1.27%)	
Other	336,713 (46.51%)	7,580 (53.46%)	
Insurance provider			< 0.01
Medicare	201,623 (27.85%)	5,611 (39.57%)	
Medicaid	205,098 (28.33%)	4,230 (29.83%)	
Private	245,856 (33.96%)	3,264 (23.02%)	
Self-pay	71,382 (9.86%)	1,075 (7.58%)	
Charlson comorbidity index			< 0.01
0	272,136 (37.59%)	0 (0%)	
1	230,798 (31.88%)	3,785 (26.69%)	
2	105,843 (14.62%)	3,175 (22.39%)	
3 or more	115,182 (15.91%)	7,220 (50.92%)	
Median Income in patient zip code			< 0.01
$ 1 - $ 51,999	228,771 (31.60%)	4,949 (34.90%)	
$ 52,000 - $ 65,999	191,777 (26.49%)	3,956 (27.90%)	
$ 66,000 - $ 87,999	172,157 (23.78%)	3,137 (22.12%)	
> $ 88,000	131,254 (18.13%)	2,138 (15.08%)	
Hospital region			< 0.01
Northeast	122,060 (16.86%)	2,284 (16.11%)	
Midwest	165,642 (22.88%)	3,096 (21.83%)	
South	288,787 (39.89%)	6,140 (43.30%)	
West	147,470 (20.37%)	2,660 (18.76%)	
Hospital location/teaching status			< 0.01
Rural	78,043 (10.78%)	1,340 (9.45%)	
Urban nonteaching	146,891 (20.29%)	2,450 (17.28%)	
Urban teaching	499,025 (68.93%)	10,390 (73.27%)	
Hospital size			< 0.01
Small	201,985 (27.90%)	3,460 (24.40%)	
Medium	211,396 (29.20%)	4,155 (29.3%)	
Large	310,578 (42.90%)	6,565 (46.30%)	
Race			< 0.01
White	451,461 (62.36%)	9,010 (63.54%)	
Black	127,489 (17.61%)	2,181 (15.38%)	
Hispanic	101,716 (14.05%)	2,110 (14.88%)	
Asian or Pacific Islander	16,289 (2.25%)	312 (2.20%)	
Native American	6,805 (0.94%)	188 (1.33%)	
Other	20,199 (2.79%)	379 (2.67%)	
Comorbidity			
CVA	941 (0.13%)	10 (0.07%)	0.39
Congestive heart failure	43,365 (5.99%)	1,680 (11.85%)	< 0.01
Diabetes mellitus type 1	8,905 (1.23%)	187 (1.32%)	0.98
Diabetes mellitus type 2	184,681 (25.51%)	5,580 (39.35%)	< 0.01
Chronic kidney disease	62,622 (8.65%)	2,260 (15.94%)	< 0.01
Obesity	150,945 (20.85%)	2,864 (20.20%)	0.41
Smoker/nicotine dependence	243,178 (33.59%)	4,924 (34.73%)	0.21

Patients without LC had a higher prevalence of a history of cerebrovascular accident (0.13% vs. 0.07%, P = 0.39) and obesity (20.85% vs. 20.20%, P = 0.41). Patients with LC had a higher prevalence of congestive heart failure (CHF) (11.85% vs. 5.99%, P < 0.01), diabetes mellitus type 2 (DM2) (39.35% vs. 25.51%, P < 0.01), diabetes mellitus type 1 (DM1) (1.32% vs. 1.23%, P = 0.98), chronic kidney disease (CKD) (15.94% vs. 8.65%, P < 0.01), and smoking/tobacco use (34.73% vs. 33.59%, P = 0.21).

In-hospital mortality

The total all-cause in-hospital mortality for all patients with AP was 4,429 (0.6%). It was 184 (1.3%) in AP patients with LC and 4,324 (0.6%) in AP patients without LC. AP patients with LC had a higher likelihood of in-hospital mortality as compared to patients without cirrhosis (aOR: 1.74, 95% CI: 1.22-2.42, P < 0.01) when adjusted for confounders using multivariate logistic regression analysis, and this difference was statistically significant.

Length of hospital stay

The mean LOS in all patients admitted with AP was 4.33 days; 4.99 days in patients with LC and 4.31 days in patients without LC. Patients with LC had a longer hospital LOS as compared to patients without LC (+0.46 days, 95% CI: 0.26-0.67, P < 0.01) when adjusted for confounders using multivariate logistic regression analysis, and this difference was statistically significant.

Total hospitalization charges

The mean total hospitalization charges for all patients admitted with AP were $47,884, $54,686 for patients with LC, and $47,751 for patients without LC. Patients with LC had higher total hospitalization charges as compared to patients without LC (+$4,163, 95% CI: 1530.9-6796.0, P < 0.01) when adjusted for confounders using multivariate logistic regression analysis, and this difference was statistically significant.

Acute renal failure

The overall incidence of acute renal failure (ARF) in all patients admitted with AP was 97,213 (13.17%). It was 2,795 (19.71%) in AP patients with LC and 94,404 (13.04%) in AP patients without LC. Patients with LC had a higher likelihood of developing ARF compared to patients without LC (aOR 1.44, 95% CI: 1.30-1.59, P < 0.01) when adjusted for confounders using multivariate logistic regression analysis, and this difference was statistically significant.

Sepsis

The overall incidence of sepsis in all patients with AP was 42,812 (5.8%). It was 851 (6.0%) in patients with LC and 41,990 (5.8%) in patients without LC. Patients with LC had higher odds of developing sepsis than patients without cirrhosis (aOR: 1.02, 95% CI: 0.87-1.20, P = 0.78) when adjusted for confounders using multivariate logistic regression analysis; however, this difference was not statistically significant.

Shock

The overall incidence of shock was 7,381 (1.0%) in all patients with AP. It was 312 (2.2%) in patients with LC and 7,240 (1.0%) in patients without LC. AP patients with LC had a higher likelihood of developing ARF compared to patients without LC (aOR: 1.87, 95% CI: 1.42-2.46, P < 0.01) when adjusted for confounders using multivariate logistic regression analysis, and this difference was statistically significant.

ICU admission

The total incidence of ICU admission in patients with AP was 25,097 (3.4%). It was 908 (6.4%) in patients with LC and 24,615 (3.4%) in patients without LC. AP patients with LC had a higher likelihood of requiring ICU admission when compared to patients without LC (aOR 1.67, 95% CI: 1.38-1.91, P < 0.01) when adjusted for confounders using multivariate logistic regression analysis, and this difference was statistically significant.

Venous thromboembolism

The total incidence of VTE in patients with AP was 11,072 (1.5%). It was 355 (2.5%) in patients with LC and 10,859 (1.5%) in patients without LC. AP patients with LC had a higher likelihood of developing VTE when compared to patients without liver cirrhosis (aOR 1.65, 95% CI: 1.30-2.11, P < 0.01) when adjusted for confounders using multivariate logistic regression analysis, and this difference was statistically significant.

## Discussion

AP is one of the most common causes of GI-related diseases that require hospital admission [1]. The national incidence of AP is rising due to current lifestyle trends such as an increase in alcohol intake, high-fat diet consumption, and increased prevalence of obesity and gallstone disease [12]. This study investigated the impact of chronic LC on the outcomes among patients hospitalized with AP.

Our study shows that patients with AP and LC had worse overall clinical outcomes when compared to AP patients without LC. We believe one of the major reasons for this outcome was the high prevalence of comorbidities like DM2, CHF, DM1, and CKD in AP patients with LC than in patients without LC. As a result, patients with LC had a higher overall CCI and higher morbidity and mortality outcomes [13-15].

We found increased odds of ARF in patients with LC and AP. In AP, kidney injury is most likely from a pre-renal etiology due to intravascular volume depletion from pancreatic inflammation causing extravasation of intravascular fluid [5]. Patients with preexisting LC are already prone to hypoalbuminemia and the third spacing of intravascular fluid. Patients with LC are also susceptible to splanchnic arterial vasodilatation, causing a reduction in effective circulating blood volume and renal perfusion, causing hepatorenal syndrome or acute tubular necrosis [16,17]. Hence, when AP occurs in patients with LC, they have a higher likelihood of developing ARF than patients who only have AP without coexisting LC [18].

We noticed increased odds of VTE in patients with LC. The liver plays an important role in producing proteins that regulate blood clotting. When the liver is damaged in cirrhosis, it cannot synthesize enough of these anticoagulant proteins, leading to a pro-coagulant state that favors blood clot formation. This imbalance in clotting factors, combined with factors like slowed blood flow due to portal hypertension, further increases the risk of VTE [19]. AP leads to an acute inflammatory response in the body that further increases the risk of developing VTE. Hence, when patients with LC who are already at risk for VTE develop an insult like AP, we believe it increases the odds of developing VTE.

We noticed an increased risk of shock and need for ICU admission in AP patients with LC. People with LC have a significantly higher risk of developing septic shock due to impaired immune function caused by the damaged liver tissue, which leads to increased bacterial translocation from the gut, making them more susceptible to infections that can rapidly progress to severe sepsis and septic shock; this is further exacerbated by the compromised ability of the cirrhotic liver to clear bacteria and toxins from the bloodstream [20]. In LC, the splanchnic arterial vasodilatation causes a reduction in effective circulating blood volume, and coexisting AP causes systemic inflammation leading to increased vascular permeability, causing third spacing of intravenous fluids. This can lead to severe intravascular volume depletion, causing hypotension and eventually leading to shock and multi-organ failure. Hence the reason we believe that AP patients with LC were more prone to requiring ICU admission and developing shock [21].

We found AP patients with LC to have higher overall in-hospital mortality. We believe patients with LC had a higher CCI and a higher prevalence of comorbid conditions such as CHF, CKD, DM2, and DM1, causing an increased risk of mortality. LC itself leads to an immunosuppressed status, preventing the liver from performing its vital functions, leading to complications like ascites, variceal bleeding, hepatic encephalopathy, and increased susceptibility to infections, ultimately causing liver failure and death if left untreated. The cirrhotic liver cannot properly filter toxins from the blood, making patients with cirrhosis highly vulnerable to complications that can be fatal [22]. We noted higher odds of ARF, VTE, shock, and ICU admission for patients with LC. We believe all of these factors likely caused a higher mortality in AP patients with LC.

We found LOS and total hospitalization charges to be higher in AP patients with LC. We believe that the increased odds of complications such as ARF, VTE, shock, ICU admission, and mortality all contributed to longer LOS and higher total hospitalization charges in AP patients with LC. Several studies have reported an increased prevalence of infections, the need for antibiotics, and interventions in AP patients with LC, all causing increased LOS and hospitalization cost utilization [23-26].

We believe our study is important as it highlights the higher inpatient mortality and overall poor outcomes of AP in patients with LC as compared to patients without LC. It alerts physicians to appropriately risk stratify these patients and to closely monitor them for worse clinical outcomes. AP patients with LC need a multidisciplinary approach for treatment and management of AP likely involving hepatology service for the management of liver cirrhosis, nephrology service in case ARF is suspected, and infectious disease service in case infection is suspected. These early interventions can be useful to optimize the care of cirrhotics hospitalized with AP.

Our study has certain shortcomings. We used the NIS database, which uses ICD-10 codes to generate diagnoses. Incorrect or erroneous ICD-10 codes for AP or LC might have been added for patients who did not have AP, which could have skewed the data. However, due to the large sample size of the database, we believe this would not significantly affect the overall results. We used a multivariate regression model to adjust for confounders, including diverse patient and hospital-level characteristics, instead of randomization, which cannot be performed using NIS. Our study is retrospective and only establishes associations and does not establish causality. The NIS database does not allow us to stratify the severity of AP or the severity of LC. We can hence not stratify our outcomes based on the severity of AP or LC.

Our study has several strengths. Our study includes a large sample size and a wide geographic distribution of patient population taken from the NIS database. The results generated from our data can be generalized to the U.S. population as a whole, as the NIS is a stratified representative sample of the U.S. population. This increases the power of the study and renders it less prone to selection bias. This is superior to many other epidemiologic studies where data is included from small geographic areas like a single state or multicenter study results, which cannot be nationally generalized. Our study was also strengthened by the use of a multivariate regression analysis that adjusted for numerous potential confounding factors. The study was also adjusted for age, gender, race, CCI, median income quartile, and hospital characteristics.

## Conclusions

We found increased mortality and overall worse clinical outcomes of AP in patients with LC. Physicians should take extra caution in managing AP in cirrhotics. We believe more prospective multicenter studies are needed to corroborate our findings and improve the generalizability of our findings.
